# Interlinked Microcone Resistive Sensors Based on Self-Assembly Carbon Nanotubes Film for Monitoring of Signals

**DOI:** 10.3390/nano12142325

**Published:** 2022-07-06

**Authors:** Chun-Li Luo, Jun-Yi Jiao, Xing-Jie Su, Lin-Xin Zheng, Wei-Guo Yan, Dong-Zhou Zhong

**Affiliations:** 1 School of Control and Mechanical Engineering, Tianjin Chengjian University, Tianjin 300384, China; luocl@tcu.edu.cn (C.-L.L.); junyi_0614@163.com (J.-Y.J.); abcsxj666@163.com (X.-J.S.); zlx620189@163.com (L.-X.Z.); 2 School of Science, Tianjin Chengjian University, Tianjin 300384, China; 3 Intelligent Manufacturing Faculty, Wuyi University, Jiangmen 529020, China

**Keywords:** template transfer, interlinked structure, Langmuir–Blodgett, self-assembly, flexible

## Abstract

Flexible pressure sensors still face difficulties achieving a constantly adaptable micronanostructure of substrate materials. Interlinked microcone resistive sensors were fabricated by polydimethylsiloxane (PDMS) nanocone array. PDMS nanocone array was achieved by the second transferring tapered polymethyl methacrylate (PMMA) structure. In addition, self-assembly 2D carbon nanotubes (CNTs) networks as a conducting layer were prepared by a low-cost, dependable, and ultrafast Langmuir–Blodgett (LB) process. In addition, the self-assembled two-dimensional carbon nanotubes (CNTs) network as a conductive layer can change the internal resistance due to pressure. The results showed that the interlinked sensor with a nanocone structure can detect the external pressure by the change of resistivity and had a sensitive resistance change in the low pressure (<200 Pa), good stability through 2800 cycles, and a detection limit of 10 kPa. Based on these properties, the electric signals were tested, including swallowing throat, finger bending, finger pressing, and paper folding. The simulation model of the sensors with different structural parameters under external pressure was established. With the advantages of high sensitivity, stability, and wide detection range, this sensor shows great potential for monitoring human motion and can be used in wearable devices.

## 1. Introduction

Components capable of sensing external signals, for instance an external force, are essential equipment of flexible electronics skins [1,2,3,4]. Therefore, the preparation and performance improvement of various types of flexible sensors are important research directions for electronic instance skin [5,6,7,8,9,10]. The resistive sensor, which translates an external force into electrical resistance variation, has received a lot of attention because of its benefits of simple construction, easy signal reading methods, and low-cost device manufacture [11]. However, how to further improve the sensitivity and reliability of the flexible resistive pressure sensor remains a challenge. The use of microstructure in the design of flexible devices is critical to increasing sensitivity [12,13,14,15,16]. Therefore, in recent years, the interlinked microstructure sensor has inspired the interest of many academics [17]. For example, Lu et al. [18] used interlinked nanocone arrays to prepare flexible sensors sensitive to pressure response with a sensitivity of 268.36 kPa${}^{-1}$ in the pressure range of 0–200 Pa. Niu et al. [19] submitted a capacitive sensor for tactile detection based on an interlinked asymmetric nanocone array, which has several remarkable advantages, including high sensitivity (6.583 kPa${}^{-1}$) in the low-pressure region (0–100 Pa), an ultra-low detection limit (≈3 Pa), and outstanding stability and reproducibility (10,000 cycles). At the moment, practically all interlinked sensors require the use of molds to recreate the surface structure while fabricating micronanostructured films. Molds are classified into two types: silica [20,21,22,23] and natural biological materials [24,25,26]. Silica templates require traditional lithography techniques, and their structures can be adjusted at micron scales, such as microdomes, micropyramids, and microcolumns. However, the manufacture of silicon templates is time-consuming, device-dependent, and costly. Natural biological materials including rose leaves [27], lotus leaves [28], mimosa leaves, and butterfly wings [29] may be used directly to recreate minuscule designs. However, due to the intrinsic properties of a microstructure, adjusting its geometric parameters is challenging.

Selecting an excellent conducting layer is an important factor in fabricating highly sensitive flexible resistive pressure sensors with interlinked microcone sturctures. CNTs exhibit good conductivity, low toxicity, good ductility, and low cost, which makes them attractive candidate materials for wearable strain/pressure sensors [30]. Wu et al. [31] successfully fabricated an aerogel containing CNTs for a piezoresistive pressure sensor, which has good mechanical properties, compressibility, and high sensitivity (4.97 kPa${}^{-1}$ in 0–3 kPa and 0.05 kPa${}^{-1}$ in 40–80 kPa). Tang et al. [32] prepared a CNT electronic textile vacuum pressure sensor which showed good linearity, repeatability, and durability, and the maximum sensitivity is 3.06. Taak et al. [33] reported a new field emission pressure sensor via embedded CNTs, which has the advantages of high sensitivity and excellent detection dynamic range. How to efficiently prepare the large area and high-density CNT networks is still a challenge.

In this paper, a flexible interlinked resistive sensor with the microstructures was fabricated by the second template transferring method. In addition, self-assembly 2D carbon nanotubes (CNTs) networks, as a conducting layer, were prepared by a low-cost, dependable, and ultrafast Langmuir–Blodgett (LB) process. The interlinked sensors exhibited a sensitive resistance change in the low pressure (<200 Pa), high stability under repeated loading (2800 cycles), and a wide working pressure range (0–10 kPa). Moreover, the mathematical model of the interlinked flexible resistive pressure sensor was established, and the stress distribution of the sensor under external pressure was simulated. Finally, the interlinked resistive sensor was used for the detections of throat swallowing, finger bending and pressing, and paper folding.

## 2. Experimental Section

### 2.1. Materials

The polystyrene (PS) microspheres with 1 μm diameter were provided by Huge Biotechnology Co., Ltd. Shanghai, China. PMMA plate was purchased from Merck Co., Ltd. NY, USA. The mixed solution of PS microspheres and alcohol was put in a closed plastic U-shaped tube into an ultrasonic cleaner (Jie Kang, PS-20A) and shaken for 5 min. PDMS was purchased from Dow Corning (Sylgard 184). Experimental reagents were weighed with an electronic analytical balance (AR124CN) manufactured by Shanghai Shangping Instrument Co., Shanghai, Ltd., China. A 20–200 uL adjustable micro single-channel pipette was provided by Shandong Boke Scientific Instrument Co., Ltd. Shandong, China. Multi-walled CNTs (inner diameter 3–5 nm, outer diameter 8–15 nm, length 3–12 µm) were provided by Beijing Deke Daojin Science And Technology Co., Ltd. Beijing, China. Double-sided conductive copper foil tape was used as a lead to connect sensor and test equipment.

### 2.2. Preparation of the Monolayer PS Spheres Array

First, the glass was cleaned with a deionized water and alcohol solution repeatedly and finally put into the blast drying oven to dry the glass. To improve the surface hydrophilicity of the glass, the substrate was cleaned with oxygen plasma for 3 min. This plasma cleaning system was supplied by YZD08-5C Plasma Cleaner. The suspension of monodisperse PS microspheres (ethanol concentration of 5%) was treated with ultrasound for 5 min, and the microspheres were evenly dispersed in the solution. The prepared glass substrate was placed flatly on self-assembly equipment, and a certain amount of deionized water was dropped on the surface. Then the PS sphere suspension was injected on the water and self-assembled to the PS array with a large area and a monolayer. Then, the edge of the cleaned PMMA was clamped with tweezers and immersed in water. Then the monolayer PS film was transferred to the surface of the PMMA plate.

### 2.3. Fabrication of the PDMS Film with Convex Microcone

**Figure 1:**
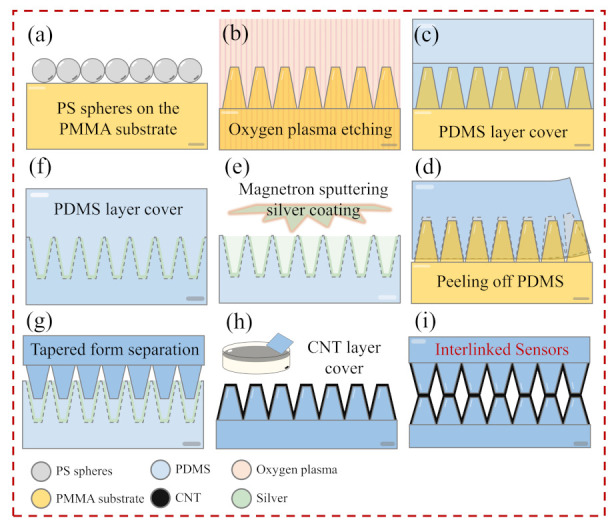
The scheme of fabricating the interlinked resistive sensors: (**a**) self-assembly of PS microspheres on PMMA substrate; (**b**) microcone structure was prepared by oxygen plasma etching; (**c**) PDMS was coated on the microcone structure; (**d**) PDMS layer on the microcone film was stripped; (**e**) the silver layer was deposited on the PDMS microporous template by magnetron sputtering; (**f**) PDMS was coated on the microporous template; (**g**) PDMS film with tapered structure was stripped; (**h**) CNTs were coated on the microcone PDMS film; (**i**) interlock sensor was combined by two microcone films.

A PMMA cones array was obtained by oxygen plasma etching of the PMMA plate with PS spheres array for 6 min. The PDMS mixed with a curing agent was poured on the PMMA cones array and then allowed to solidify for 1 h at 80 °C. The cured PDMS film was peeled away from the PMMA microcone substrate and formed a concave microhole array. To achieve the separation of the PDMS template, 30 nm thick silver as an anti-blocking layer was deposited by magnetron sputtering.The silver layer can prevent the two layers of cured PDMS layer fusion. As a stripping agent, it helps form the upper PDMS conical structure. The PDMS microcone template coated with Ag film was coated with the PDMS mixture for a second time to prepare the microcone array. The cured PDMS was again peeled away from the PDMS microhole template to yield a PDMS film with a convex microcone as shown in Figure 1.

### 2.4. Preparation of the Monolayer CNTs Networks

The mixed solution of CNTs and ethanol with a proper volume was dripped onto the water surface in the vessel. There are two key factors: (1) moderate drop speed and (2) the volume of mixed solution. A moderate drop speed is required to produce a homogeneous CNTs films on the water surface. When the injection volume of mixed solution approaches saturation, the phenomenon of agglomeration became more and more prominent. As a consequence, homogeneous CTNs film were eventually created on the liquid/air interface by suitable injection. Then, a surfactant such as detergent was selected and placed on one side of the interface, and then the Langmuir area decreased significantly. It is worth noting that the homogeneous Langmuir monolayer is closely stacked on the side opposite the active agent. When the film’s move behavior stops and additional surfactant cannot drive the film, the resulting film is generated, exhibiting a densely packed structure.

### 2.5. Manufacture of Flexible Pressure Sensor

To achieve a flexible pressure sensor with interlinked microcone structure, the PDMS microcone structure was transferred from the same template. The PDMS film (2 cm × 2 cm) with microcone was coated with CNTs film by transferring the monolayer CNTs film from the water surface to the PDMS substrate by the LB method. The resistance of the CNTs/PDMS surface was about 30–70 kΩ. Then, the pressure sensor was assembled by the interlock of the upper and lower membrane. The copper strip was used as the test electrode, and the conductive layer was bonded with conductive adhesive. The interlinked sensor was composed of interlinked microstructure PDMS films.

### 2.6. Characterization and Instruments

The plasma cleaning system was supplied by YZD08-5C Plasma Cleaner. The plasma etching system was supplied by Jiangsu Leuven Instrument Co., Ltd. Pizhou, China. The silver layers were prepared using a three-target magnetron sputtering instrument (VTC-600-3HD). The morphology of the microstructure was characterized by field emission scanning electron microscopy (FESEM) (Ultra Plus, Zeiss). The component of CTNs was analyzed by Raman spectroscopy (532 nm laser source, XploRA, HORIBA Jobin Yvon). The resistance was measured by UNI-T UT 8.04 million with a meter. A semiconductor parameter analyzer (Keithley, 4200A-SCS) was used to determine the sensor’s current-voltage (I-V) properties.

### 2.7. Simulation Analysis of Interlinked Sensor

To analyze the relationship between the pressure and the contact area of the sensor, a 3D model was built by COMSOL Multiphysics software. The geometric parameters of the model were consistent with the size of SEM images.

## 3. Results and Discussion

### 3.1. Sensor Design and Fabrication

Figure 1 shows the scheme of fabricating the interlinked resistive sensors. To achieve the microcone arrays of PDMS, the second transferring process was needed in our experiments. Firstly, the PMMA coated with a single layer PS microsphere array was etched by oxygen plasma to obtain the microcone structure template. Secondly, PDMS liquid was uniformly coated on the PMMA template by the drop-coating method, and the concave template was obtained after curing and stripping. Then, a layer of nanosilver was plated on the PDMS template by magnetron sputtering as an anti-adhesion layer, and the microcone structure PDMS substrate was obtained by secondary transfer. Finally, the CNTs thin films prepared by the highly simplified LB method were transferred to the flexible substrate, and the flexible pressure sensor was obtained by interlinked nanocone arrays.

**Figure 2:**
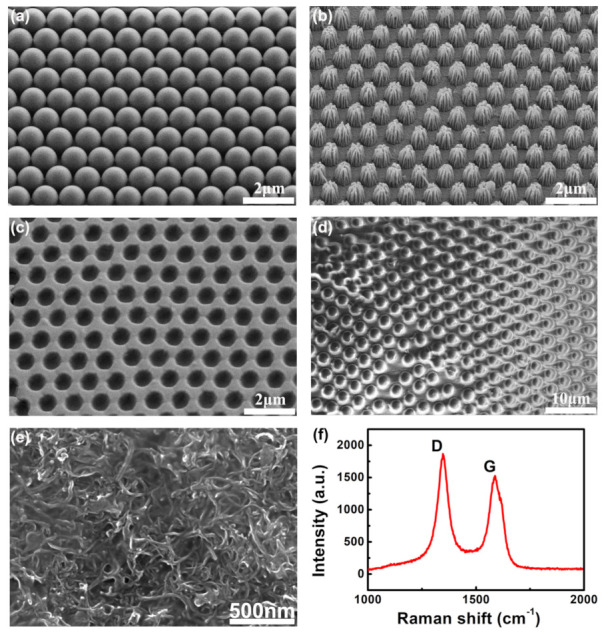
The SEM of fabricating flexible pressure sensor during different processes and Raman spectra of CNTs: (**a**) the monolayer PS spheres arrays on PMMA substrate; (**b**) the PS microcone array was etched with oxygen plasma after 6 min; (**c**) the SEM image of concave PDMS film by the first transferred from the PMMA microcone array template; (**d**) the PDMS’ SEM image with microcone structure; (**e**) the SEM image of the CNTs networks covered on the PDMS substrate; (**f**) Raman spectra of CNTs.

The self-assembly monolayer PS microspheres exhibit a honeycomb structure on the PMMA substrate, as illustrated in Figure 2a. According to the minimum energy principle, the close-packed honeycomb PS structure remains stable during the self-assembly process. The PS array was etched by oxygen plasma for 6 min to form a cone-shaped array with a slightly wider bottom and a slightly narrower top on the PMMA substrate, as shown in Figure 2b. The choice of etching time depends on the size of the PS microspheres and the etching power. The taper of the PMMA is affected by etching time, and an insufficient etching time will increase the retention rate of PS microspheres, which is not conducive to the formation of the PMMA taper. Conversely, too long an etching time will reduce the height and yield of the microcone. In the experiment, the PS/PMMA template was etched for 6 min, and an ordered microcone array was formed on the PMMA surface while the PS microspheres were completely removed.Then we covered the PMMA cone array with PDMS, and peeled off the cured PDMS from the PMMA microcone substrate. Figure 2c shows the SEM image of the surface of the PDMS layer with a uniform funnel-shaped. These uniformly inverted PDMS holes indicated that the PMMA microcone array was completely embedded in the PDMS surface. Figure 2d shows the SEM image of the regular and very uniform microcone array on the PDMS, which was successfully fabricated by the second transferring method. Figure 2e is the SEM image of the CNTs networks on the PDMS microcone surface. It can be seen from the image that the network structure of CNTs is compact, so it has very high stability. As shown in Figure 2f, there are two obvious Raman peaks at around 1334 and 1585 cm${}^{-1}$, which correspond to the D band and G band of CNTs, respectively. Using CNT networks as a conductive layer for microstructure sensors can significantly improve the stability and durability, thus avoiding material degradation and loss of accuracy caused by frequently repeated deformation.

### 3.2. Sensor Model Development

**Figure 3:**
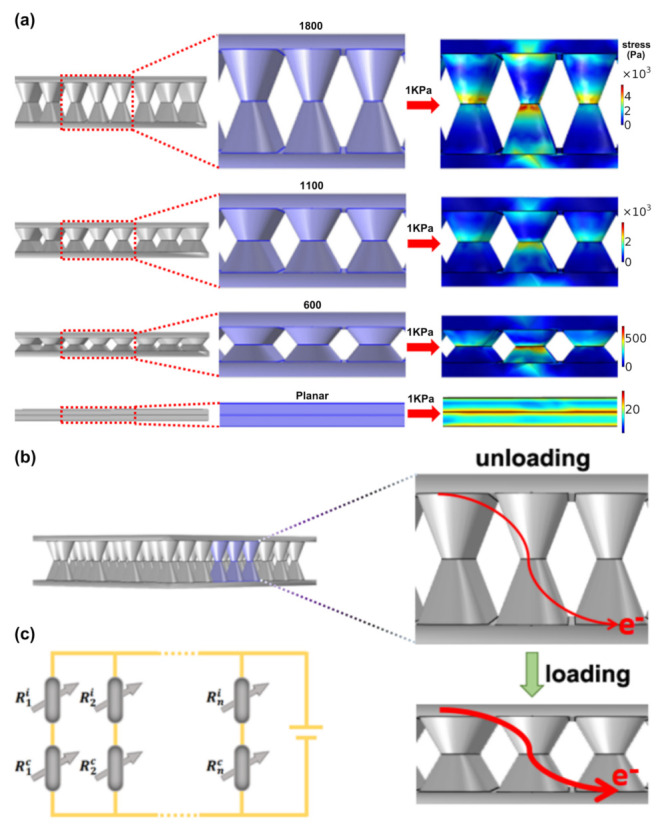
(**a**) The simulation shows the local stress distribution of the interlinked resistance sensor under an applied pressure of 1 kPa; (**b**) schematic diagram of the sensing mechanism under loading and unloading processes; (**c**) equivalent circuit diagram of the interlinked resistive sensor.

For the pressure of 1 kPa, Figure 3a demonstrates the local stress change of the emulated interlinked highly ordered microcone array and interlinked conductive planar sheet. Here, the heights of the sensor single layer microcone are set to 1800 nm, 1100 nm, and 600 nm and then defined as sensor-1800, sensor-1100, and sensor-600, respectively. The heights of sensor-1800, sensor-1100, and sensor-600 are 3.6, 2.2, and 1.2 μm, respectively. The simulated results show that the contact area between interlinked microcones increases, and the height decreases when the applied load increases. In addition, the local stress distribution is concentrated on the contact surfaces between the interlinked microcones. However, the interlinked planar film without microcone array does not exhibit the phenomena of stress concentration. From the simulated results, we can see that sensor-1800 obviously has a wider stress distribution than sensor-1100 and sensor-600. Figure 3b shows the structural schematic drawing of the interlinked microcone array under normal and loaded conditions. The interlinked microcone array’s reaction to stress is proportional to its height and the contact area between the microcones. In this situation, the contact resistance ($R^{c}$) and the intrinsic resistance ($R^{i}=2r^{i}$) between the two face-to-face microcones constitute the resistance of the interlinked microcone array, as reflected by the resistive properties of the conductive film, which may be stated as

(1) $$R=R^{c}+R^{i}$$

The $R^{i}$ of PDMS microcones can be defined as

(2) $$R^{i}=\;\rho \frac{L}{s}$$

where ρ is the PDMS microcone resistivity, *L* is the interlinked nanocone height, and *s* is the interlinked nanocone’s equivalent cross-sectional area.

According to the Holm equation [34], $R^{c}$ is inversely proportional to the contact area (A) between microcones, which expressed as

(3) $$R^{c}\;=\frac{1}{2k\sqrt{A/\pi }}$$

where *A* denotes the contact area between the interlinked microcones, and *k* denotes the conductivity of the two contacting microcones. Therefore,

(4) $$R=\frac{1}{2k\sqrt{A/\pi }}+\rho \frac{L}{s}$$

The equivalent-circuit schematic of the interlinked microcone is shown in Figure 3c. According to the equivalent-circuit schematic, the resistance circuit of the interlinked microcone is in parallel mode.

(5) $$\begin{array}{cc} R^{\sigma } & =R_{1}^{\sigma }//{\;R}_{2}^{\sigma }//\cdots //{\;R}_{j}^{\sigma }//\cdots //{\;R}_{n}^{\sigma },\\ j & =1,2,\cdots ,n \end{array}$$

where $R^{\sigma }$ is the overall resistance of the interlinked microcone array under applied stress σ, $R_{j}^{\sigma }$ is the resistance value of an effective conductive channel inside the microcone array under applied stress σ, and // denotes a parallel connection

(6) $$\begin{array}{cc} R^{\sigma } & =\;{\left(R^{c}+R^{i}\right)}_{1}^{\sigma }\;//{\left(R^{c}+R^{i}\right)}_{2}^{\sigma }//\cdots //{\left(R^{c}+R^{i}\right)}_{j}^{\sigma }\;//\cdots //{\left(R^{c}+R^{i}\right)}_{n\;}^{\sigma },\\ j & =1,2,\cdots ,n \end{array}$$

Because the sensing material’s piezoresistive property is the relationship between the electrical conductivity of the interlinked microcone array and the applied external force, the sensitivity *S* can be expressed as [35,36,37,38,39,40]

(7) $$S\;=\;\frac{\Delta R/R_{0}}{P}\times 100\%$$

(8) $$\Delta R=\left|R-R_{0}\right|$$

where *P* represents the relative applied pressure and *R* and R0 represent the resistance and initial resistance, respectively.

Based on the circuit model of the interlinked microcone array, the following formulas are derived

(9) $$\frac{\Delta R}{R_{0}}=\left(1-\frac{R_{0}^{i}}{R_{0}}\right)\times \left(1-\sqrt{A_{0}/A}\right)+\frac{R_{0}^{i}}{R_{0}}\times \frac{\Delta L}{L_{0}}$$

Therefore,

(10) $$S\;=\;\frac{\Delta R/R_{0}}{P}=\left(1-\frac{R_{0}^{i}}{R_{0}}\right)\times \left(\frac{1-\sqrt{A_{0}/A}}{P}\right)+\frac{R_{0}^{i}}{R_{0}}\times \frac{\Delta L/L_{0}}{P}$$

Here, $R_{0}$ and $R_{0}^{i}$ are constants representing the starting resistance of the interlinked microcone array and the total of the body resistances ($R^{i}$) of the two interlinked nanocones, respectively. As a result, the $\Delta R/R_{0}$ is strongly connected to the ($1-\sqrt{A_{0}/A}$) and $\Delta L/L_{0}$.

### 3.3. The Performance of the Interlinked Microcone Resistive Sensor

**Figure 4:**
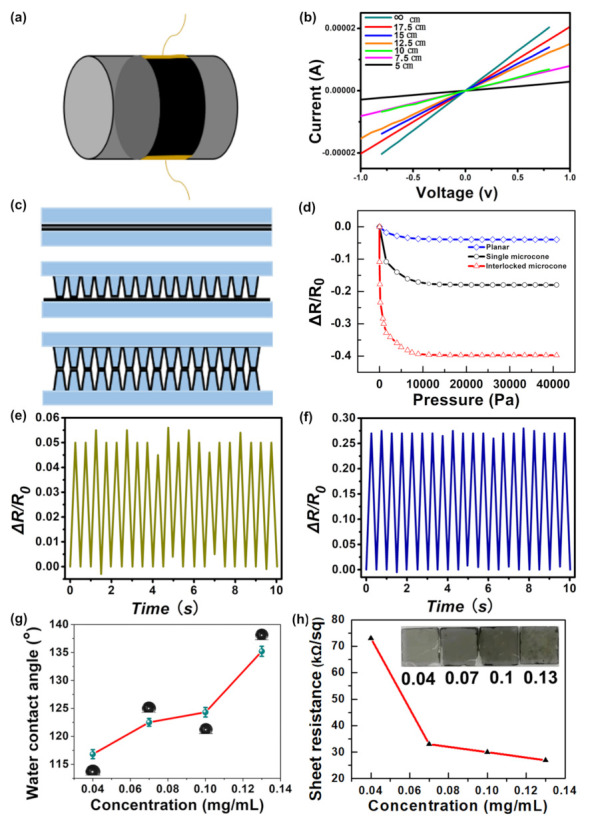
The performance test of pressure sensors: (**a**) schematic diagram of pressure sensor settled on the curved surface; (**b**) the current–voltage (I-V) curves of pressure sensor under different bending radius; (**c**) the diagram of three distinct sensor; (**d**) pressure sensitivities of three sensor topologies: planar film (blue), single microcone (black), and interlinked microcone (red), respectively; (**e**,**f**) the real-time resistance change of the sensors without microstructure and with microcone structure during cyclic bending; (**g**) the content angle of CNTs networks under different concentration; (**h**) the resistance of CNTs networks under different concentration.

As illustrated in Figure 4a, the sensor was mounted on a curved glass surface to explore the electrical properties in bending conditions. Figure 4b depicts the current–voltage (I-V) curves of the sensors at different bending degrees. While the voltage changes from −1 V to 1 V, the bending radius is kept constant. From the I-V curves, it is seen that the slope of the I-V curves reduces with an increase of bending deformation. When the radius of curvature is *∞*, the current of the interlinked microcone array changes from −0.02 mA to 0.02 mA. When the radius of curvature is 5 cm, the current of the interlinked microcone array changes from −0.003 mA to 0.003 mA. The I-V curves retain excellent linearity under different bending degrees.

To evaluate the influence of the surface microcone on the resistivity, three different pressure sensors were measured, including planar without microstructure, single-layer microcone, and interlinked microcone with a double-layer (Figure 4c).The resistance variations of the sensors under the same external force is shown in Figure 4d. For all sensors, the resistance is negatively correlated with the external force, and the increase of pressure significantly reduces the resistance of the sensor. However, compared with the planar sensor, the interlinked sensor has a large resistance change, and a larger response can be observed under pressure stimulation. When the external pressure is 4000 Pa, the resistance change rate of the interlinked microcone sensor is −35.6%, which is significantly higher than that of the planar sensor, −2.8%, and the single microcone sensor, −14%. When the applied pressure is in the 4000–40,000 Pa range, the sensor resistance drops rather slowly and tends to be steady as the pressure increases. The results show that the sensitivity of the interlinked microstructure sensor is significantly higher than that of the sensor with a planar surface because the microstructure generates a larger contact area due to deformation under the same external load, resulting in lower contact resistance between the two interlinked conductive films. We summarized previous research and compared them in Table 1.

Figure 4e depicts the pressure sensor’s response curve without the microstructure during the cyclic bending process, whereas Figure 4f depicts the sensor’s real-time resistance variations with the microcone structure. The resistance of the latter changes more significantly during the bending process. It was discovered that the resistances of the film increased or decreased in proportion to the deformation of the finger. Furthermore, the films did not detach after being subjected to joint movements, owing to their high adhesive ability. These findings suggest that the film demonstrated steady and sensitive conductivity during dynamic activities, indicating that it might have applications in soft human-motion sensors for electrical signal monitoring.

The contact angle of water, as shown in Figure 4g, characterizes the hydrophobic behavior of CNTs films formed with varied initial CNTs dispersion concentrations. The contact angle of the CNT film prepared by 0.13 mg/mL dispersion was larger than 135°, which indicates that it has strong hydrophobicity. The conductive films formed by self-assembly of CNT dispersions with different concentrations are shown in Figure 4f. The increase of concentration caused the decrease of the resistance. When the concentration of the dispersion was increased from 0.04 mg/mL to 0.07 mg/mL, the resistance of the formed films was significantly reduced from 73 to 32 kΩ/sq. Therefore, the pressure sensor based on CNTs has broad application prospects, such as signal monitoring in a humid environment, wearable medical monitoring equipment, and electronic skin.

### 3.4. The Specific Application of the Interlinked Microcone Resistive Pressure Sensor

**Figure 5:**
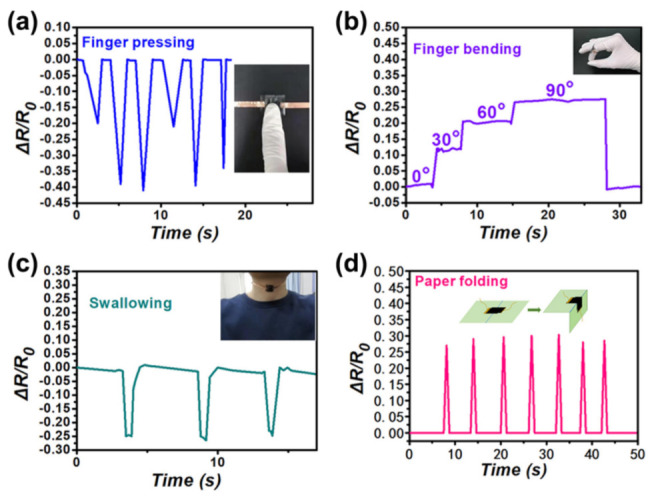
(**a**) The measurement of a finger pressing, inset: photo of finger pressing pressure sensor; (**b**) finger bending produces responsive curves (0°, 30°, 60°, and 90°), illustration: photo of pressure sensor fixed to finger; (**c**) detection of motion in the human larynx during swallowing; (**d**) the sensor’s resistance varies when it is fixed in the folds of cardboard.

The rapid response of the interlinked sensor to external force is conducive to its detection of the random finger pressing. Figure 5a depicts the dynamic resistance fluctuation in response to stochastic finger contact, illustrating the interlinked sensor’s ability to detect and discriminate between different amounts of pressure. On a finger push, the resistance abruptly reduces and quickly recovers to its initial resistance once the finger is withdrawn. Following that, the sensor’s reaction to finger bending was examined, which has an obvious reference for possible applications in robotics. The flexible interlinked sensor can be attached to human fingers to detect different motion states. It was discovered that the bending condition of the finger could be recognized, such as 0°, 30°, 60°, and 90°. A significant resistance shift was noticed, which was determined to be related to mechanical strain (Figure 5b). The monitoring result of swallowing activity by the interlinked sensor is shown in the figure (Figure 5c). The sensor’s outstanding distinguishing ability of laryngeal motion was demonstrated by the three typical troughs produced. Such sensors might be used in a physiological monitor, alerting caregivers to any possible concerns. To comprehensively test the capabilities of the sensor, the experiment of surface folding was also carried out (Figure 5d). When the cardboard was bent at 90°, the resistance changed dramatically. Because of the prominent peak, the measurement curve revealed that the sensor had an instantly detectable ability. The above test results show that the interlinked microstructure flexible resistive pressure sensor has the function of monitoring various signals and has a wide application prospect in flexible electronic devices.

**Figure 6:**
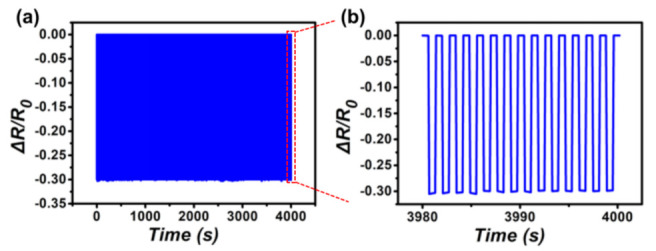
(**a**) Relative resistance change of the sensor when 2800 cycles of loading/unloading are performed at 1 kPa external pressure; (**b**) the 14 cyclic tests extracted from the red region in (**a**).

As shown in Figure 6a, to test the stability of this sensor, 2800 cycles of loading and unloading tests were performed with an external force of 1 kPa. During the continuous loading and unloading cycle, the amplitude and waveform of the relative resistance change signal were highly similar, and no drift was found, which proved the high durability of the sensor. Figure 6b explains that the relative resistance remains constant after 2800 cycles. The interlinked microcone array structure gives the sensor high stability; therefore, it can withstand frequent mechanical deformation.

## 4. Conclusions

In conclusion, the flexible resistive pressure sensor based on the PDMS interlinked microstructures was fabricated by the second template transferring method. In addition, 2D CNTs networks, as a conducting layer, were prepared by the simple LB method to overcome the fracture in the process of bending metal film. The interlinked sensors with microcones exhibit a sensitive resistance change in the low pressure (<200 Pa), good stability through 2800 cycles, and a detection limit of 10 kPa. Furthermore, the sensor signals of throat swallowing, finger pressing, finger bending, and paper folding were measured accurately. This pressure sensor based on the PDMS interlinked microstructures with simple preparation, low cost, and high sensitivity has a wide application prospect in flexible wearable electronic devices.

## Figures and Tables

**Table 1:** Comparison of the sensitivity based on this work and previous reports.

Reference	Sensitivity (kPa${}^{-1}$)	Pressure Range (kPa)	Sensing Mechanism
17	0.034	25	Capacitive
18	0.055	10	Capacitive
19	0.55	7	Capacitive
20	1.12	40	Capacitive
21	15	5	Resistance
This work	5.4	10	Resistance

## Data Availability

Data presented in this article are available at request from the corresponding author.

## Sample Availability

Samples of the compounds are available from the authors.
